# Supplementary material for: ATP5B Is an Essential Factor for Hepatitis B Virus Entry
Source: Int J Mol Sci. 2022 Aug 24;23(17):9570. doi: 10.3390/ijms23179570 (PMC9455612; doi:10.3390/ijms23179570)
Supplement: Supplementary file 1 [file ijms-23-09570-s001.zip › ijms-1862638-supplementary.pdf]

Supplementary information.

Materials and Methods.

Determination of the interaction region of ATP5B with myrPS1:2-47. In order to verify the direct interaction of myrPS1: 2-47 with ATP5B, we tried to express and purify ATP5B in E. coli. An N-terminally streptavidin-tagged ATP5B with a histidine hexamer at the C-terminus expression vector was constructed using a pQE trisystem His-Strep 2 vector (Quiagen) according to the manufacturer's instructions. In this construct, a streptavidin-GFP-His expression vector was constructed. We constructed the deletion mutants such as ATP5B: 1-198aa, 1-397aa, 1-275aa, 199-266 and 163-529aa expression versions in the same vector. Unfortunately, ATP5B: 1-397aa, 1-275aa and 199-266 were not expressed well in E. coli and thus these versions were converted to mammalian expression constructs using pFN21A (Promega), in which Halo®-tag was added at the N-terminus of each expression vector. The Halo-tagged ATP5B: 1-397aa, 1-275aa and 199-266 as well as the full-length construct (1-529aa) and Halo-tag only were transfected into HEK293T cells with LT-I™ transfection reagent according to the manufacturer's instructions.

Full ATP5B and its deletion mutants, 1-198aa, 163-529aa and GFP, were induced for expression with 1 mM IPTG at 0.6 ~ 0.7 of OD630 and cultured for a few additional hours. Then, the cells were harvested and suspended in a Nickel-NTA (Ni-NTA) buffer (20 mM $NaPO_4$, pH 8.0, 0.3 M NaCl, 10 % glycerol, 10 mM 2-mercaptoethanol and 0.1% Triton® X-100), lysozyme was added at 1 mg/ml, and Benzonase® nuclease (Novagen) was added at 3 U/ml. After incubating on ice for 30 min, the cells were disrupted with sonication five times for 20 seconds each with 30 second intervals on ice. The cleared lysate was obtained by centrifugation followed by passage through a 0.45 μm filter unit (Millipore™). The filtrated sample was purified with Nickel NTA agarose and eluted with 0.5 M imidazole. The purified sample was adjusted at 0.1 mg/ml and an aliquot (~1 ml) was incubated with biotinylated myrPS1 peptide at 4˚C overnight and purified with streptavidin Sepharose™. After washing with the Nickel-NTA (Ni-NTA) buffer, the bound fraction was eluted with 100 μl of 1x sample buffer (62.5 mM Tris-HCl, pH 6.8, 1% SDS, 10% glycerol, 0.02% bromophenol blue and 70 mM 2-mercaptoethanol) and boiled for 10 min. The input and the bound fraction were separated on an SDS-PAGE and subjected to Western blotting analysis. A mouse monoclonal anti-Strep tag antibody (Quiagen) was used for detection along with anti-mouse IgG conjugated with horseradish.

In the case of mammalian expression, the transfected cells were harvested a few days post-transfection and lysed with the Ni-NTA buffer. The cell debris was removed by centrifugation and the lysate was passed through a 0.45 μm filter unit (Millipore). After taking some input sample, the lysate was then incubated with biotinylated myrPS1: 2-47 or PS2: 2-55 overnight at 4˚C. After centrifugation, the unbound fraction was stored and the bound fraction was washed three times with the buffer, eluted with 100 μl of 1x sample buffer and boiled for 10 min. The input, the unbound and the bound fraction were subjected to an SDS-PAGE followed by Western blotting analysis. A mouse monoclonal anti-Halo antibody (Promega) was used for detection as described above.

Chemiluminescence was generated with a chemiluminescence substrate (SuperSignal™ West Pico Chemiluminescence Substrate; Pierce) according to the manufacturer's instructions, and the signal was detected with a ChemiDoc™ MP imager (BioRad).

HBV infection blocking assay with ATP5B peptides. After determination of the interaction region on ATP5B, several peptides around the interaction region—i.e., ATP5B: 160-210aa, 195-240aa, 195-250aa and 225-250aa—were synthesized by the manufacturer to test which region was really responsible for the interaction. These peptides were added to the medium at concentrations of 0.01, 0.1 and 1 μM only at the infection. After infection, the inoculum was washed away and then the medium was refreshed. The medium was refreshed every 3 days. MyrPS1 peptide was used for inhibition control. Nine days after starting the experiment, the medium was harvested and the eAg was evaluated with an e Antigen ELISA Kit (Bioneovan Co.). Data were shown as the mean value with the standard deviation.

Isolation of shATP5B clones. Poly clonal shATP5B cells were seeded 200 cells in 10 cm dish (AGC Techno Glass Co., LTD). After colonies grew, about twenty colonies were isolated in 24 well plate (AGC Techno Glass Co., LTD) with a penicillin cup. When the well was full of cells, the cells were harvested and subjected to Western blot analysis with a rabbit anti-ATP5B antibody (Sigma) or a mouse anti-GAPDH antibody (Sigma) followed by anti-mouse IgG conjugated either with IRDye® 680LT or 800CW (LI-COR®) according to the orientation. Isolated clones such as #15, #16, #17 and #18 were maintained and tested again for knockdown of ATP5B and then HBV infectivity as described in the context.

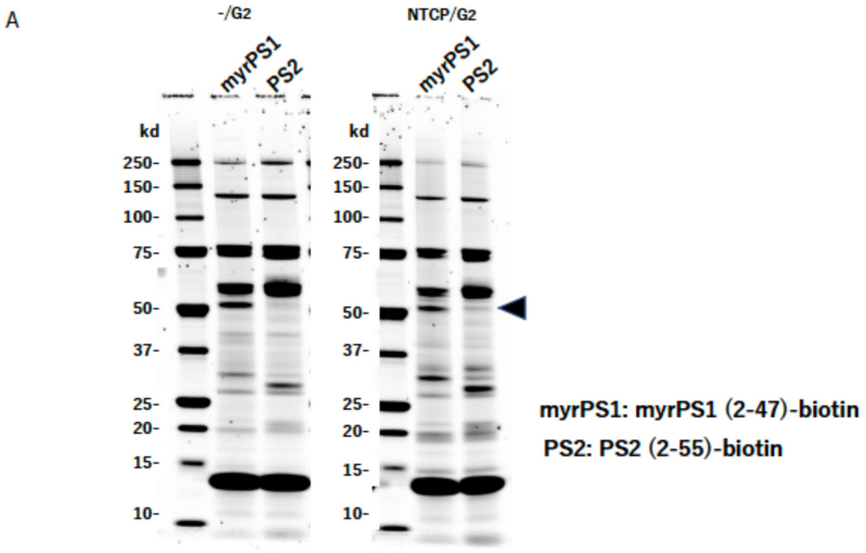

B

ATP5B (ATP synthase ß)

IPI00303476 (100%), 56,560.6 Da
ATP5B ATP synthase subunit beta, mitochondrial
12 exclusive unique peptides, 17 exclusive unique spectra, 21 total spectra, 191/529 amino acids (36% coverage)

```
MLGFVGRVAA APASGALRRL TPSASLPPAQ LLLRAAPTAV HPVRDYAAQT
SPSPKAGAAT GRIVAVIGAV VDVQFDEGLP PILNALEVQG RETRLVLEVA
QHLGESTVRT IAMDGTEGLV RGQKVLDSGA PIKIPVGPET LGRIMNVIGE
PIDERGPIKT KQFAPIHAEA PEFMEMSVEQ EILVTGIKVV DLLAPYAKGG
KIGLFGGAGV GKTVLIMELI NNVAKAHGGY SVFAGVGERT REGNDLYHEM
IESGVINLKD ATSKVALVYG QMNEPPGARA RVALTGLTVA EYFRDQEGQD
VLLFIDNIFR FTQAGSEVSA LLGRIPSAVG YQPTLATDMG TMQERITTTK
KGSITSVQAI YVPADDLTDP APATTFAHLD ATTVLSRAIA ELGIYPAVDP
LDSTSRIMDP NIVGSEHYDV ARGVQKILQD YKSLQDIIAI LGMDELSEED
KLTVSRARKI QRFLSQPFQV AEVFTGHMGK LVPLKETIKG FQQILAGEYD
HLPEQAFYMV GPIEEAVAKA DKLAEEHSS
```

Figure S1. Pull-down assay to identify myrPS1 interacting factors. A pull-down assay was performed as described in the Materials and Methods of the main text. A. Separated proteins stained with Coomassie Brilliant Blue on the SDS-PAGE. A preS2 peptide (PS2: 2-55) was also used for comparison. The arrowhead (◂) shows the position of ATP5B. B. Peptides of ATP5B determined by MALDI-TOF/MS analysis. The band indicated by the arrowhead (◂) on the myrPS1 lane was cut out and subjected to MALDI-TOF/MS analysis, which revealed that the band was ATP5B with high probability. This was proved by a pull-down assay followed by Western blotting (please see the main text).

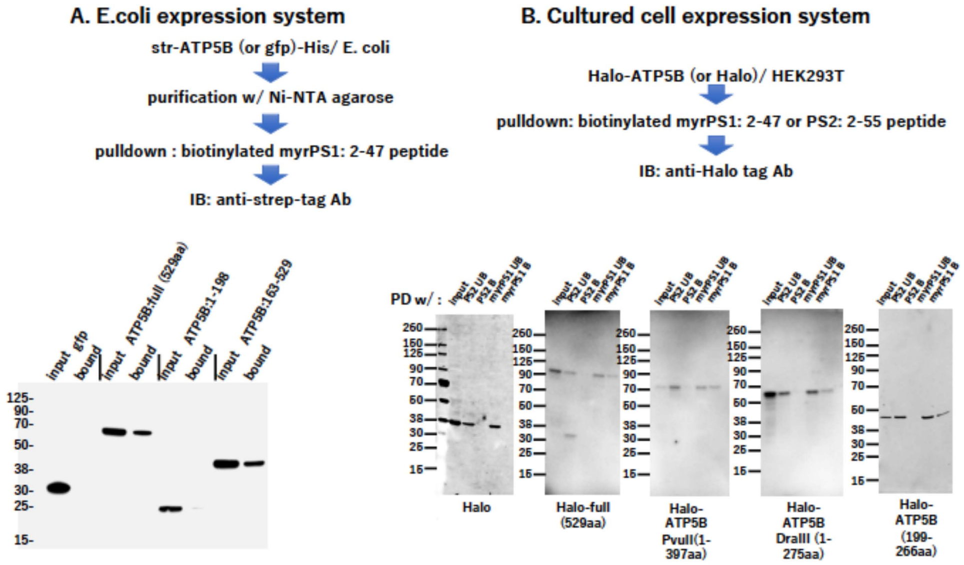

Figure S2. Determination of the interaction region of ATP5B with myrPS1. A. Determination of the interaction region by the E. coli expression system. Several parts of the ATP5B protein were successfully expressed and purified using the E. coli expression system. The expressed proteins were tagged with strep-tag at the N-terminus and His6 at the C-terminus. Input and bound fraction were separated on an SDS-PAGE and detected with an anti-strep antibody. B. Determination of the interaction region by a mammalian expression system. A Halo-tagged version of ATP5B and its deletion mutants were constructed and transiently expressed in HEK293T cells. The cell lysates were pulled down with either myrPS1: 2-47 or PS2: 2-55. The input, unbound and bound fraction were separated on an SDS-PAGE and detected with an anti-Halo antibody. The ATP-binding region of ATP5B is shown at the bottom.

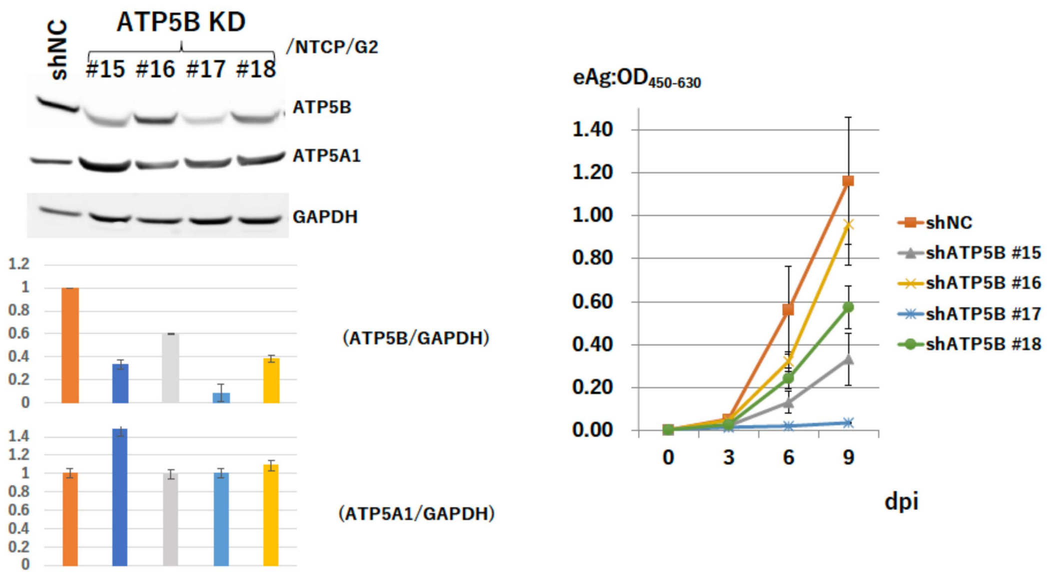

Figure S3. Isolated ATP5B KD cell clones and their infectivity to HBV. The isolated clones; #15 ~ #18 were isolated and subjected to Western blot assay (left). The level of knockdown of ATP5B was different but ATP5A1 was not reduced for expression. In the infection analysis (right), HBeAg (eAg) ELISA was performed day 0, 3, 6 and 9 post infection using the soup. The soup was diluted to one fifth. The data was shown as ELISA OD.

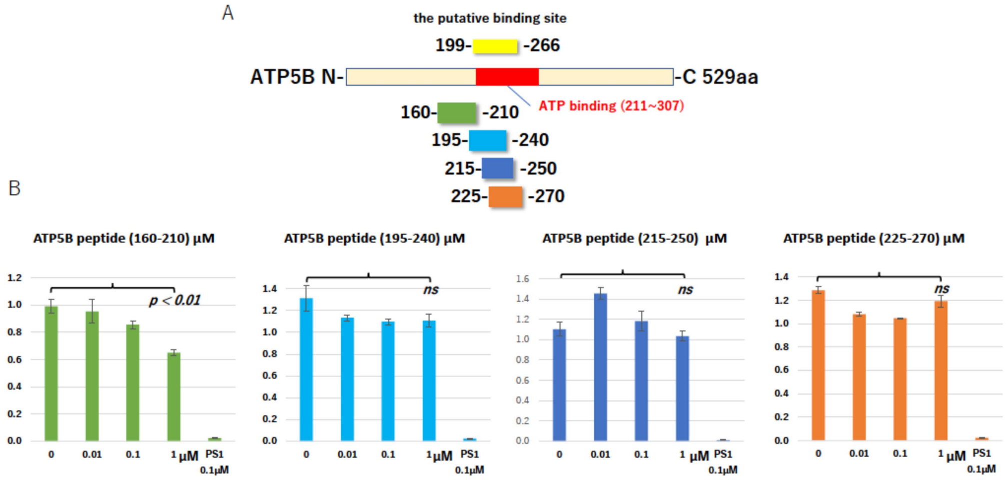

Figure S4. Peptide blocking assay of HBV infection. A. Synthesized peptides of ATP5B. Four kinds of peptide around the myrPS1 interaction region on ATP5B were synthesized. Each peptide was added only at the infection at 0, 0.01, 0.1 and 1 μM. MyrPS1 (0.1 μM) was used as a positive control for the infection block. ELISA data of eAg at the last day are shown as the mean values with the standard deviation. The infection was significantly blocked only by the highest concentration of ATP5B: 160-210aa, i.e., 1 μM ($p < 0.01$).
